# Supplementary figures and images for: Cryo-ET detects bundled triple helices but not ladders in meiotic budding yeast
Source: PLoS One. 2022 Apr 14;17(4):e0266035. doi: 10.1371/journal.pone.0266035 (PMC9009673; doi:10.1371/journal.pone.0266035)

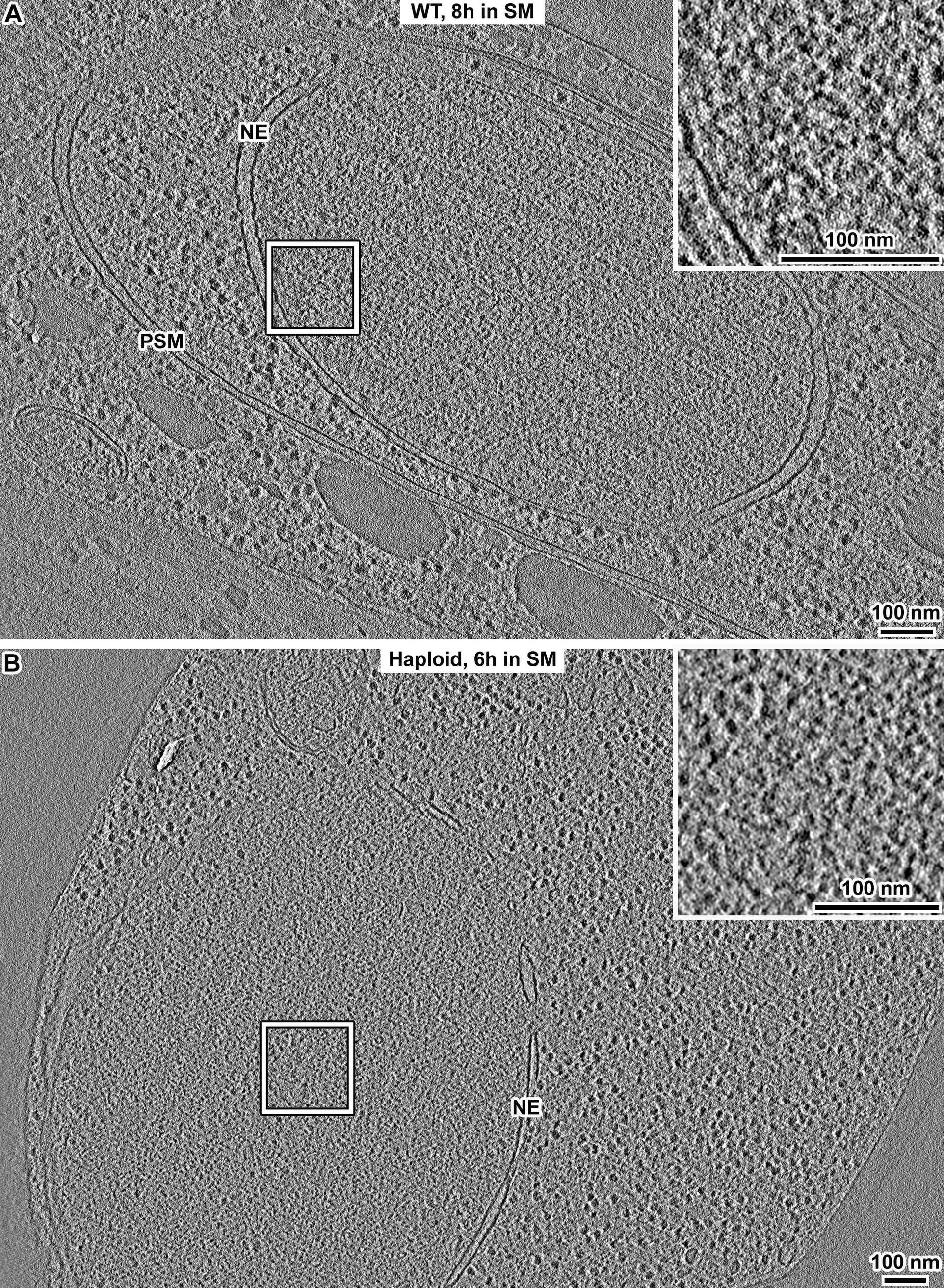

Supplement: S1 Fig — (A) Volta cryotomographic slice (12 nm; computational) of a diploid WT yeast cell after an 8-hour incubation in SM. PSM, prospore membrane; NE, nuclear envelope. (B) Volta cryotomographic slice (12 nm; computational) of a haploid cell (strain LY2, W303 background) incubated 6 hours in SM. The insets show 3-fold enlargements of the nucleoplasm boxed in each panel. (TIF) [file pone.0266035.s001.tif]

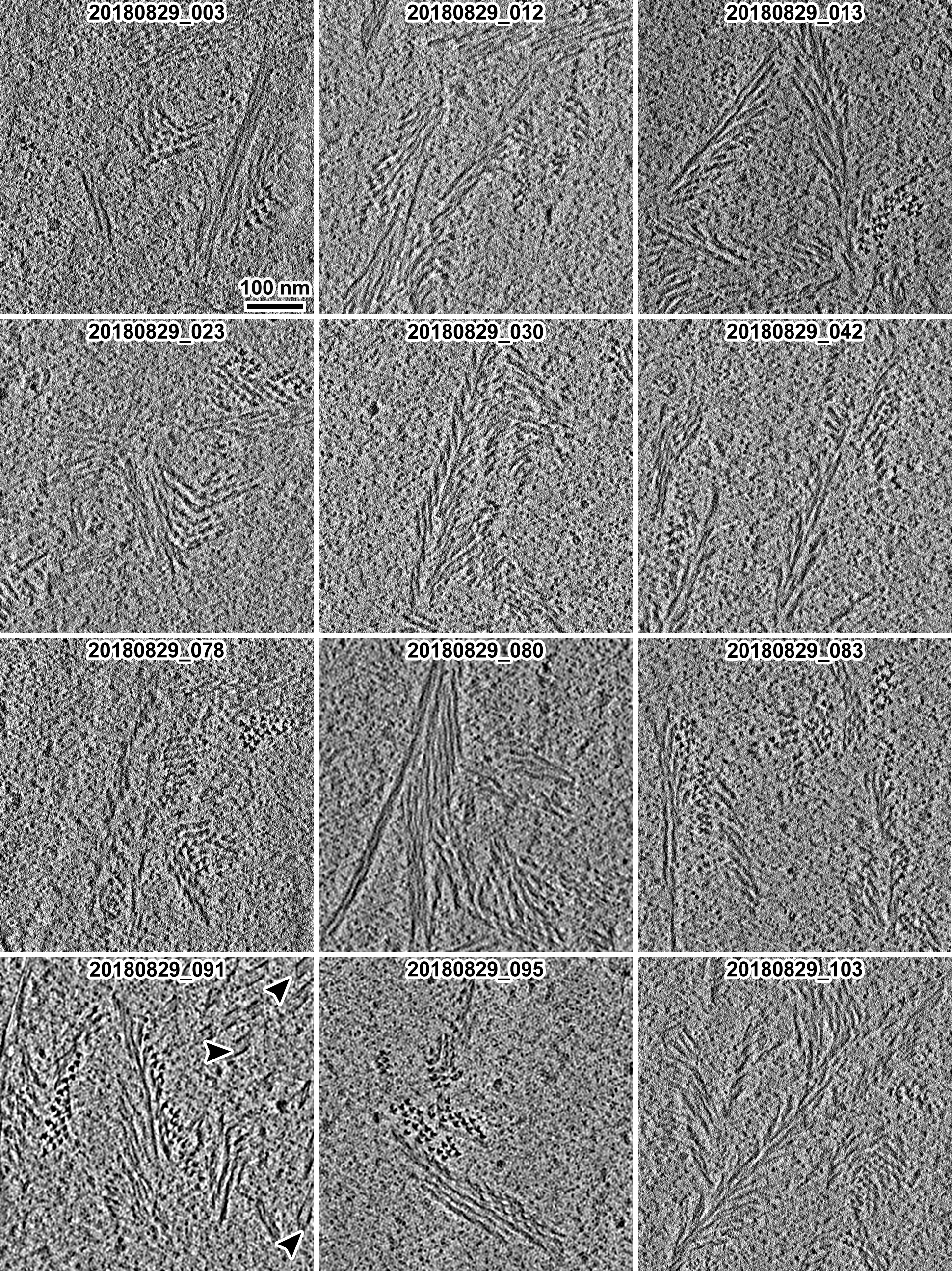

Supplement: S2 Fig — Volta cryotomographic slices (10 nm; computational) of 12 examples of ndt80Δ cell nuclei after 8 hours in SM. The arrowheads in the lower-left panel indicate nuclear microtubules. (TIF) [file pone.0266035.s002.tif]

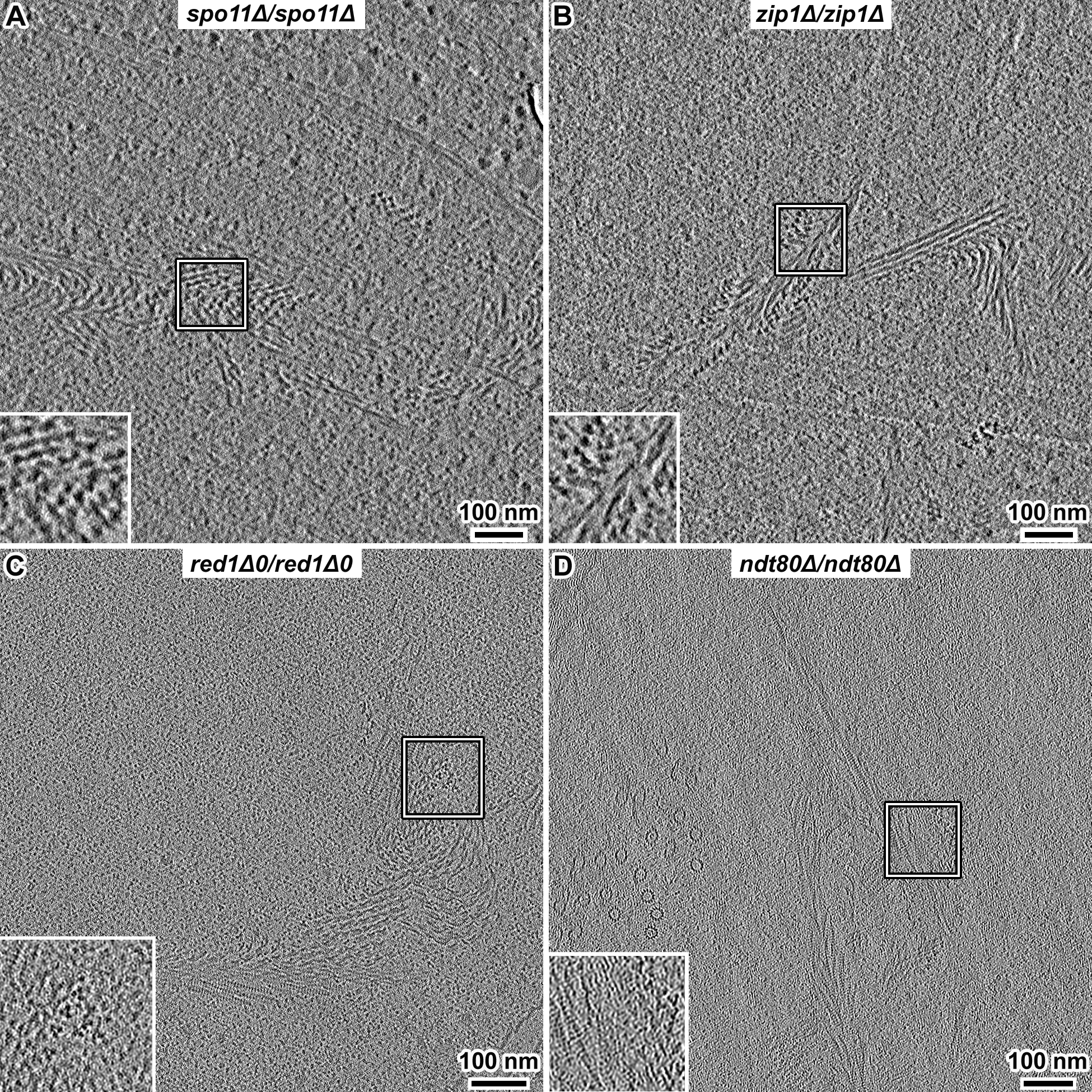

Supplement: S3 Fig — (A) Volta cryotomographic slice (6 nm; computational) of a spo11Δ cell in SM, showing intra-nuclear MTH bundles. (B) Volta cryotomographic slice (12 nm; computational) of a zip1Δ cell in SM, showing MTH bundles inside the nucleus. (C) Defocus phase-contrast cryotomographic slice (12 nm; computational) of a red1Δ0 cell in SM, showing intra-nuclear MTH bundles. Insets show two-fold enlargements of the boxed areas. The contrast in panel C appears different from panels A+B because of the different contrast mechanism (defocus phase contrast instead of Volta phase contrast) and because the data was recorded on an electron-counting camera and by zero-loss energy filtering. This difference in contrast is also evident in (D) the ndt80Δ control cell cryotomogram, imaged in similar conditions as for panel C. (TIF) [file pone.0266035.s003.tif]

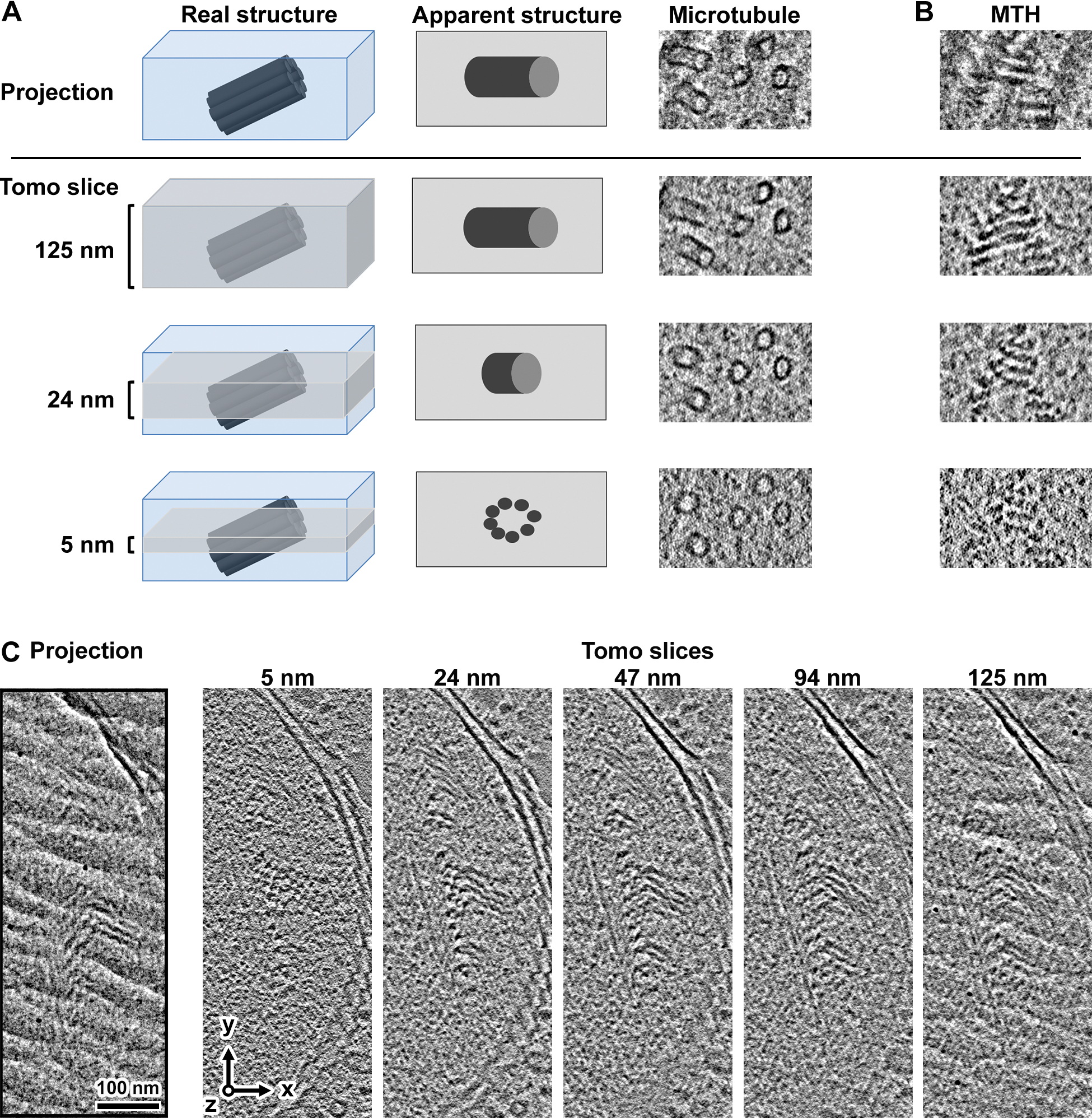

Supplement: S4 Fig — (A) Cartoon of comparison between projection and tomographic slices (computational) of various thicknesses. Thinner tomographic slices reveal details like microtubule protofilaments. Thicker tomographic slices resemble projection images. (B) In some projections, contiguous MTH structures appear to orient approximately perpendicular (left-right) to the orientation of the filaments (out of the plane). (C) An MTH bundle position in a cell that has a chevron-like motif in projection. The thinner tomographic slices (computational) show that the underlying structure is a set of densely packed MTHs that are oblique relative to the Z axis. (TIF) [file pone.0266035.s004.tif]

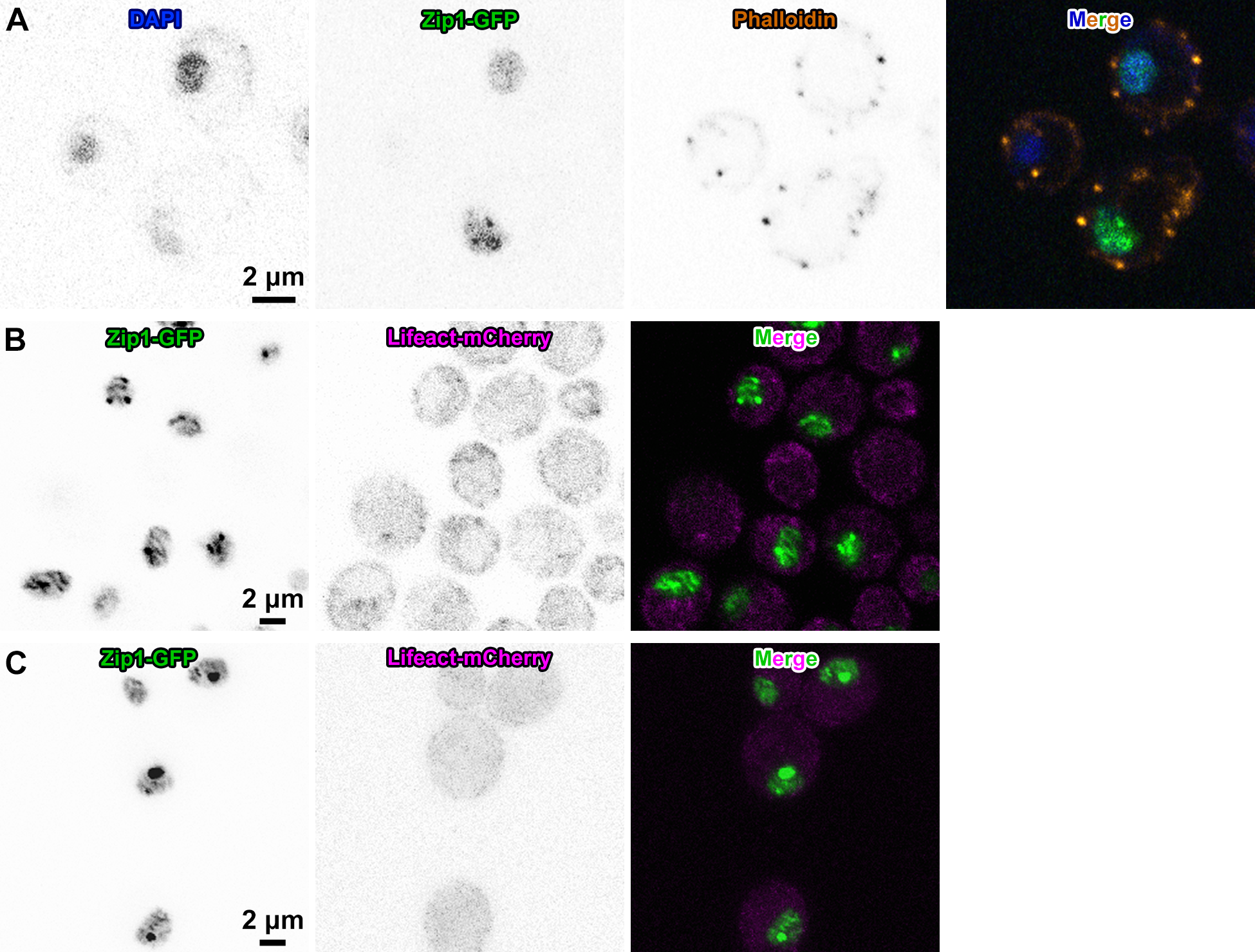

Supplement: S5 Fig — (A) Fluorescence micrographs of pachytene-arrested EW104 cells after 6 hours in SM. SCs are marked by Zip1-GFP. Phalloidin-stained F-actin does not co-localize with DAPI-stained chromatin or Zip1-GFP. (B) Confocal slices of pachytene-arrested LGY0069 cells, which express Zip1-GFP (green) and Lifeact-mCherry (magenta). (C) Maximum-intensity projections through the nuclei of LGY0069 cells. (TIF) [file pone.0266035.s005.tif]

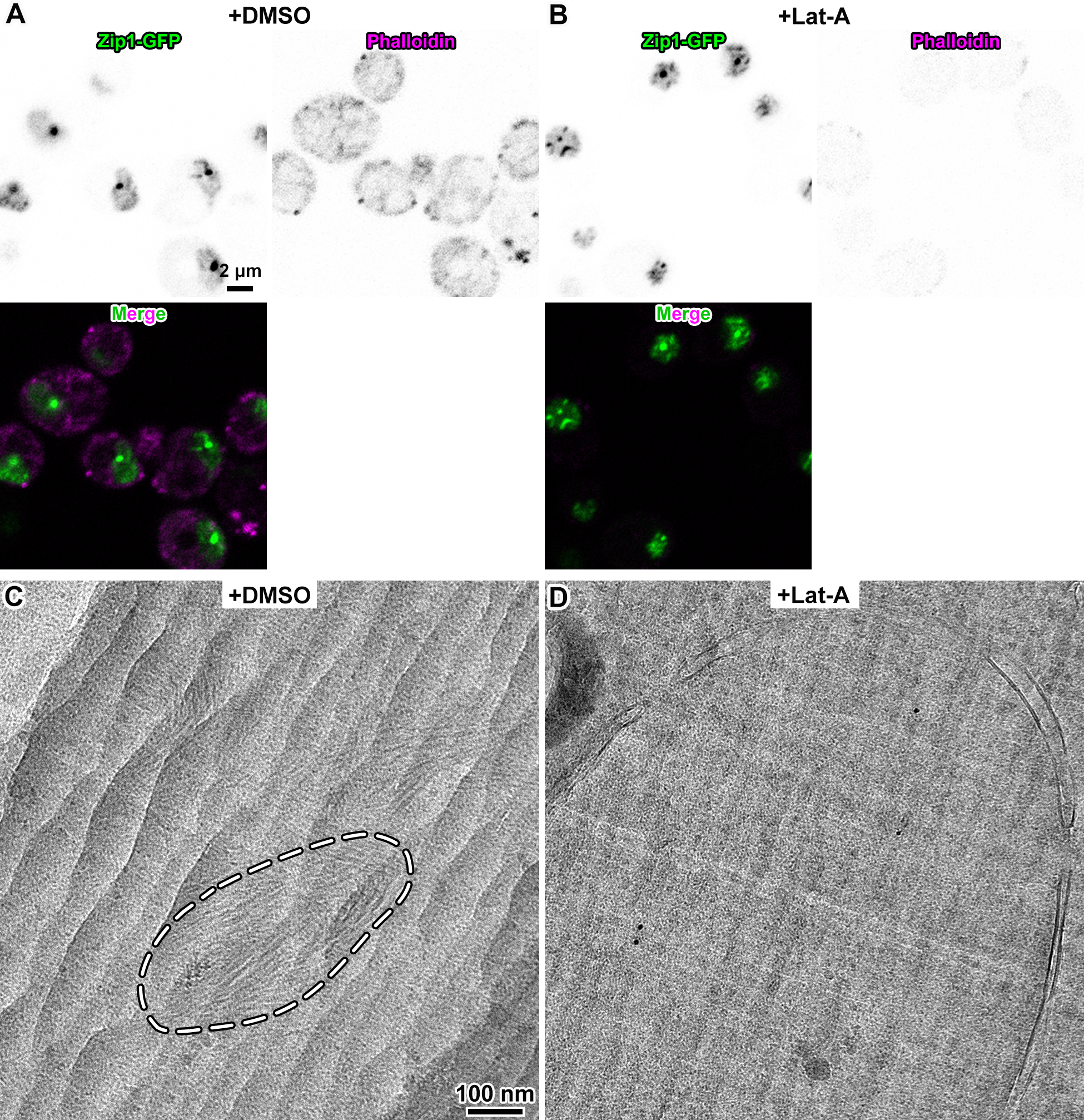

Supplement: S6 Fig — Fluorescence microscopy of (A) DMSO-treated and (B) Latrunculin A-treated EW104 (ndt80Δ) pachytene cells. (C) Projection cryo-EM image of a DMSO-treated EW104 cell, showing MTH bundles inside the nucleus. One set of MTH bundles is enclosed in the dashed oval. (D) Projection cryo-EM image of a Latrunculin A-treated EW104 ndt80Δ cell. In panels C and D, the linear pattern running along the diagonal form the upper left to the lower right is from knife marks. In panel C, the wavy patterns running along the 2 o’clock to 8 o’clock diagonal are from cryosection crevassing. These image features are not devitrification artifacts; they are absent from the tomographic slices (computational) in other figures because they can be computationally excluded. 15 of 24 cryosections of DMSO-treated cells contain MTH bundles while 0 out of 66 cryosections of Lat-A-treated cells contain MTH bundles. All cells were incubated in SM for 6 hours prior to drug treatment. See also Table 2. (TIF) [file pone.0266035.s006.tif]

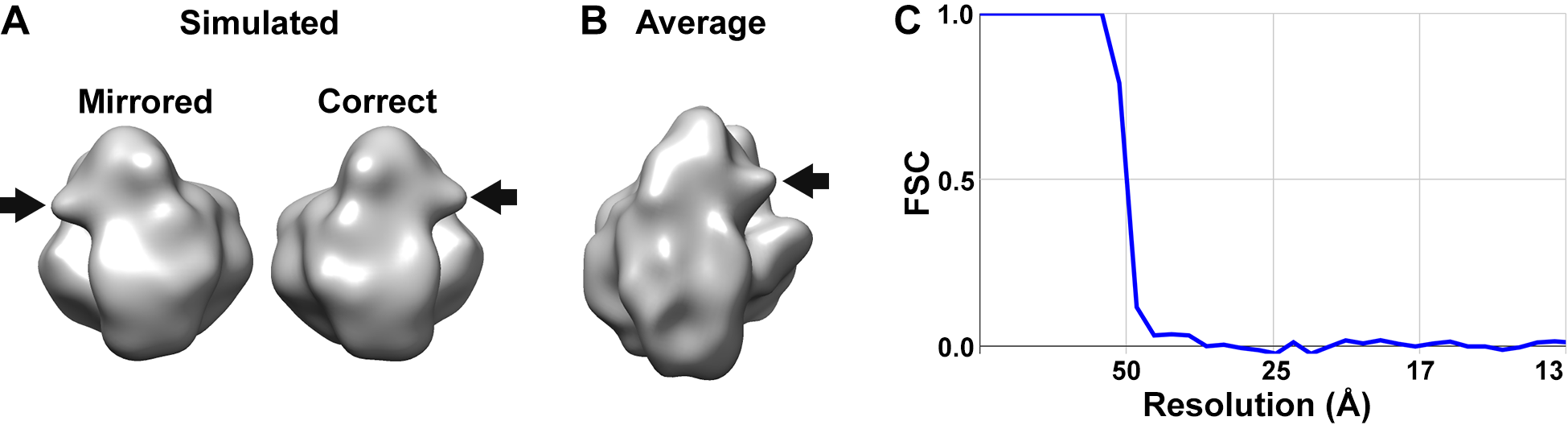

Supplement: S7 Fig — (A) Density map of the 80S yeast ribosome [97], simulated at 35 Å resolution with the wrong handedness (mirrored) and correct one. The “beak” motif is indicated by the arrow. (B) Subtomogram average of cytoplasmic ribosomes from ndt80Δ cells. (C) Fourier shell correlation (FSC) plot of the subtomogram average in panel B. The resolution is ~ 50 Å, based on the “Gold standard” FSC = 0.5 criterion. (TIF) [file pone.0266035.s007.tif]

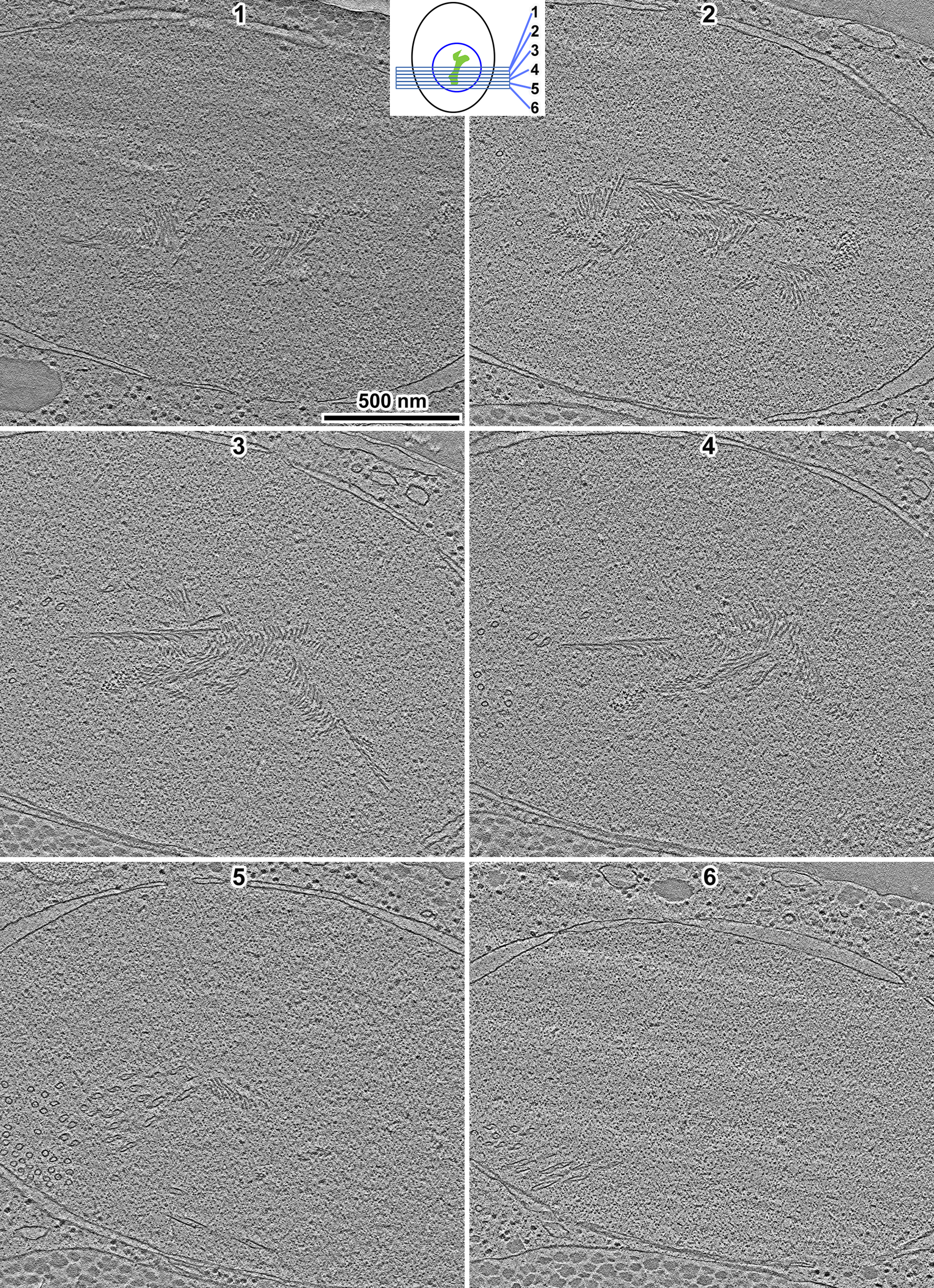

Supplement: S8 Fig — Volta cryotomographic slices (computational) of six sequential cryosections of a single ndt80Δ cell, isolated after 8 hours in SM. The schematic (not to scale) shows the relationship between the numbered cryosections and the cytology. Major features are the cell nucleus (blue circle) and the MTH bundles (green blob). The MTHs are in the center of the nucleus while the spindle is anchored at the lower left, with many nuclear microtubules visible in cryosections 4, 5, and 6. (TIF) [file pone.0266035.s008.tif]
