# Supplementary material for: Cryo-ET detects bundled triple helices but not ladders in meiotic budding yeast
Source: PLoS One. 2022 Apr 14;17(4):e0266035. doi: 10.1371/journal.pone.0266035 (PMC9009673; doi:10.1371/journal.pone.0266035)
Supplement: S1 Table — (DOCX) [file pone.0266035.s010.docx]

**S1 Table. Strains used.**

| **Strain** | **Parent** | **Genotype** | **Source** |
| --- | --- | --- | --- |
| NKY611 | - | SK1; diploid | [18] |
| DK428 * | - | *Zip1::GFP/Zip1::GFP* | [21] |
| EW104 | NKY2292, NKY2293 | *ndt80Δ::LEU2/ndt80Δ::LEU2 Zip1::GFP/Zip1::GFP* | [20] |
| LY2 | - | W303; haploid | Lacefield |
| NKY2535 | - | *spo11Δ::hisG/ spo11Δ::hisG ndt80Δ::LEU2/ndt80Δ::LEU2* | [18] |
| NKY2460 | - | *zip1Δ::LEU2/zip1Δ::LEU2* | [18] |
| LGY0068 | NKY2292, NKY2293 | *ndt80Δ::LEU2/ndt80Δ::LEU2 red1Δ0::URA3/red1Δ0::KanMX* | This paper |
| LGY0069 | EW104 | *ndt80Δ::LEU2/ndt80Δ::LEU2 Zip1::GFP/Zip1::GFP ABP140/ABP140::Lifeact-yomCherry* | This paper |

* DK428 was formerly known as HW655
