# Supplementary material for: Cryo-ET detects bundled triple helices but not ladders in meiotic budding yeast
Source: PLoS One. 2022 Apr 14;17(4):e0266035. doi: 10.1371/journal.pone.0266035 (PMC9009673; doi:10.1371/journal.pone.0266035)
Supplement: S2 Table — (DOCX) [file pone.0266035.s011.docx]

**S2 Table. Primers, 5’ → 3’.**

| **Editing** |  |
| --- | --- |
| Red1Δ0 KanMX F | CATTAAAGGTAATAAGAAAAATAGAACAAAGAATTTTTAAGCACAGGACCACAAAGGGACAGCAAATACGGTGATAAGAGACATGGAGGCCCAGAATACC |
| Red1Δ0 KanMX R | CGAACATGAATAAGCTTGCCTATATTACTTTTATTAGCCATCTTAAATCTAAAAAGAATTGCGTATATGTATACTATCAGTATAGCGACCAGCATTCAC |
| Red1Δ0 URA3 F | GAATAAGCTTGCCTATATTACTTTTATTAGCCATCTTAAATCTAAAAAGAATTGCGTATATGTATACTATCAGTATAGCGACCAGCATTCACATACG |
| Red1Δ0 URA3 R | GGTAATAAGAAAAATAGAACAAAGAATTTTTAAGCACAGGACCACAAAGGGACAGCAAATACGGTGATAAGAGACATGGAGGCCCAGAATACCCTCC |
| Lifeact-yomCherry-KanMX F | TGGGTGTCGCAGATTTGATCAAGAAATTCGAAAGCATCTCAAAGGAAGAAGGTGCTGGTTTAATTAACATGG |
| Lifeact-yomCherry-KanMX R | CGTTTGATTTGAGAGGCTTTGATGAACTGTTTGTATCAACGGTAGCATCGCCCAGTATAGCGACCAGCATTCAC |
| **Validation** |  |
| Red1Δ0 VF1 | GGAAAATCCAGTCAGTTGGAAATG |
| Red1Δ0 VR1 | CCCTCGTTTTGTTAAATAAGAGTGC |
| Red1Δ0 VF2 | CAATGACAAATCACAACAGTTTTCC |
| Red1Δ0 VR2 | CAACGTCAATTGATGCACATAAATTG |
| Red1Δ0 VF3 | GAGGATTGTTTGGTAAAGGAAGAG |
| Red1Δ0 VR3 | GAATCCATTTTTGCTGCTCTTTTATG |
| Red1Δ0 VF4 | GCGAGTGATTTTGATGACGAG |
| Red1Δ0 VR4 | CTGTAACATCATTGGCAACGC |
| Lifeact-mCherry VF1 | AGAAGTGGCACTATAGCTGTG |
| Lifeact-mCherry VR1 | GTACAGTTCATCCATACCAC |
| Lifeact-mCherry VF2 | CCATGGGTAAGGAAAAGAC |
| Lifeact-mCherry VR2 | CTCTTGTTGATGCAATTCTTTGG |
|  |  |
| **Sequencing** |  |
| Red1 deletion_conf F1 | CAAAACATTCACATCATTCGTGAAG |
| Red1 deletion_conf R2 | CCGGGATCTAGAAATATTTAATTGAGG |
| URA3 within REV | ATTGTCAGTACTGATTATAATTGG |
| Red1::KanMX conf FWD2 | CCTTACCCATGGTTGTTTAT |
