# Supplementary material for: Cryo-ET detects bundled triple helices but not ladders in meiotic budding yeast
Source: PLoS One. 2022 Apr 14;17(4):e0266035. doi: 10.1371/journal.pone.0266035 (PMC9009673; doi:10.1371/journal.pone.0266035)
Supplement: S3 Table — (DOCX) [file pone.0266035.s012.docx]

**S3 Table. Cryo-ET details.**

| **Sample preparation** |  |
| --- | --- |
| EM grids | Continuous Carbon |
| Cryomicrotome | UC7/FC7 |
| Attachment device | Crion |
| Micromanipulators | Leica micromanipulator; Narishige MN-151-S |
| Cryomicrotome feed | 70 or 100 nm |
|  |  |
| **Cryo-ET data collection** |  |
| Microscope | Titan Krios |
| Energy | 300 keV |
| Camera: recording mode | Falcon II: integration  K3-GIF: super-resolution, movie frames |
| Energy filter width | 20 eV |
| Tomography software | TFS Tomo4 and SerialEM |
| Unbinned pixel size | Falcon II: 7.3Å, 5.8Å, 4.6Å  K3-GIF: 3.4Å, 4.6Å |
| Contrast mechanism | Volta phase contrast & defocus phase contrast |
| Defocus (nominal) | Volta phase contrast: −0.5 μm  Defocus phase contrast: −9 µm |
| Cumulative dose | 60 – 120 e^−^ / Å^2^ |
| Dose fractionation | 1 / cosine |
| Tilt range | ±60°, bidirectional, negative angles first; dose-symmetric |
| Tilt increment | 2° |
|  |  |
| **Cryo-ET data analysis** |  |
| Tomogram processing | IMOD 4.10.22 beta |
| Template matching | PEET 1.13.0 |
| Reference creation | Bsoft 1.8.8 |
| Mask creation | Bsoft 1.8.8, RELION 3.0.7 |
| Subtomogram analysis | RELION 3.0.7 |
| Tomogram visualization | UCSF Chimera 1.13.1, IMOD 4.10.22 beta |
| Auxiliary scripts | https://github.com/anaphaze/ot-tools |
| Calculations | Google sheets, FIJI |
| Figure/movie editing | Adobe Photoshop, Illustrator, and Premiere Pro CC |
