# Supplementary material for: Cryo-ET detects bundled triple helices but not ladders in meiotic budding yeast
Source: PLoS One. 2022 Apr 14;17(4):e0266035. doi: 10.1371/journal.pone.0266035 (PMC9009673; doi:10.1371/journal.pone.0266035)
Supplement: S4 Table — (DOCX) [file pone.0266035.s013.docx]

**S4 Table. Cryo-EM details.**

| **Dataset** | **Strain** | **SM time, treatment** | **Figures, analysis** | **Dose** | **Pixel size (Å)** | **∆f**  **(μm)** | **Cam** |
| --- | --- | --- | --- | --- | --- | --- | --- |
| **Cryotomograms** |  |  |  |  |  |  |  |
| 20180629_139 | NKY611 | 0h | 2A | 100 | 7.3 | 0.5 | FII |
| 20180816_032 | DK428 | 2h | 2B | 100 | 5.8 | 0.5 | FII |
| 20180731_029 | DK428 | 4h | 2C | 100 | 7.3 | 0.5 | FII |
| 20181204_025 | DK428 | 6h | 2D, 6A | 100 | 5.8 | 0.5 | FII |
| 20180731_087 | EW104 | 8h | 3C | 100 | 4.6 | 0.5 | FII |
| 20181204_001 | DK428 | 6h | 4B | 100 | 5.8 | 0.5 | FII |
| 20190322_5hx_017 | DK428 | 6h, +5% hx | 4C, STA | 70 | 4.6 | 0.5 | FII |
| 20181204_7hx_021 | DK428 | 6h, +7% hx | 4D | 100 | 5.8 | 0.5 | FII |
| 20200728_wsh_025 | DK428 | 6h, +7% hx, +SM wash | 4E | 100 | 5.8 | 0.5 | FII |
| 20190429_037 | NKY611 | 4h | 5A+B; 6B+D, movie S1 STA | 100 | 5.8 | 0.5 | FII |
| 20190429_076 | NKY611 | 4h | 6C, STA | 100 | 5.8 | 0.5 | FII |
| 20190109_035 | DK428 | 8h | S1A | 100 | 5.8 | 0.5 | FII |
| 20190213_020 | LY2 | 6h | S1B | 100 | 11.7 | 0.5 | FII |
| 20180829_003 | EW104 | 8h | S2 | 100 | 5.8 | 0.5 | FII |
| 20180829_012 | EW104 | 8h | S2 | 100 | 5.8 | 0.5 | FII |
| 20180829_013 | EW104 | 8h | S2 | 100 | 5.8 | 0.5 | FII |
| 20180829_023 | EW104 | 8h | S2 | 100 | 5.8 | 0.5 | FII |
| 20180829_030 | EW104 | 8h | S2, S8 #2, STA | 100 | 5.8 | 0.5 | FII |
| 20180829_042 | EW104 | 8h | S2, STA | 100 | 5.8 | 0.5 | FII |
| 20180829_078 | EW104 | 8h | S2, STA | 100 | 5.8 | 0.5 | FII |
| 20180829_080 | EW104 | 8h | S2, STA | 100 | 5.8 | 0.5 | FII |
| 20180829_083 | EW104 | 8h | S2, STA | 100 | 5.8 | 0.5 | FII |
| 20180829_091 | EW104 | 8h | S2, STA | 100 | 5.8 | 0.5 | FII |
| 20180829_095 | EW104 | 8h | S2, STA | 100 | 5.8 | 0.5 | FII |
| 20180829_103 | EW104 | 8h | S2 | 100 | 5.8 | 0.5 | FII |
| 20200325_009 | NKY2535 | 6h | S3A | 100 | 5.8 | 0.5 | FII |
| 20200618_003 | NKY2460 | 6h | S3B | 100 | 5.8 | 0.5 | FII |
| 20201214_003 | LGY0068 | 8h | S3C | 100 | 3.4 | 9 | K3 |
| 20200304_020 | EW104 | 8h | S3D | 100 | 4.6 | 7 | FII |
| 20181204_071 | DK428 | 6h | S4C | 100 | 5.8 | 0.5 | FII |
| 20180829_024 | EW104 | 8h | S8 #1, STA | 100 | 5.8 | 0.5 | FII |
| 20180829_058 | EW104 | 8h | S8 #3, STA | 100 | 5.8 | 0.5 | FII |
| 20180829_066 | EW104 | 8h | S8 #4, STA | 100 | 5.8 | 0.5 | FII |
| 20180829_075 | EW104 | 8h | S8 #5, STA | 100 | 5.8 | 0.5 | FII |
| 20180829_088 | EW104 | 8h | S8 #6 | 100 | 5.8 | 0.5 | FII |
| 20180829_037 | EW104 | 8h | STA | 100 | 5.8 | 0.5 | FII |
| 20180829_038 | EW104 | 8h | STA | 100 | 5.8 | 0.5 | FII |
| 20180829_045 | EW104 | 8h | STA | 100 | 5.8 | 0.5 | FII |
| 20180829_048 | EW104 | 8h | STA | 100 | 5.8 | 0.5 | FII |
| 20180829_085 | EW104 | 8h | STA | 100 | 5.8 | 0.5 | FII |
| 20180829_086 | EW104 | 8h | STA | 100 | 5.8 | 0.5 | FII |
| 20190429_003 | NKY611 | 4h | STA | 100 | 5.8 | 0.5 | FII |
| 20190429_007 | NKY611 | 4h | STA | 100 | 5.8 | 0.5 | FII |
| 20190429_019 | NKY611 | 4h | STA | 100 | 5.8 | 0.5 | FII |
| 20190429_020 | NKY611 | 4h | STA | 100 | 5.8 | 0.5 | FII |
| 20190429_029 | NKY611 | 4h | STA | 100 | 5.8 | 0.5 | FII |
| 20190605_040 | NKY611 | 4h | STA | 100 | 4.6 | 0.5 | FII |
| 20190605_041 | NKY611 | 4h | STA | 100 | 4.6 | 0.5 | FII |
| 20190605_044 | NKY611 | 4h | STA | 100 | 4.6 | 0.5 | FII |
| 20190605_077 | NKY611 | 4h | STA | 100 | 4.6 | 0.5 | FII |
| 20190605_084 | NKY611 | 4h | STA | 100 | 4.6 | 0.5 | FII |
| 20190605_085 | NKY611 | 4h | STA | 100 | 4.6 | 0.5 | FII |
| 20190605_086 | NKY611 | 4h | STA | 100 | 4.6 | 0.5 | FII |
| 20190605_090 | NKY611 | 4h | STA | 100 | 4.6 | 0.5 | FII |
| 20190322_5hx_001 | DK428 | 6h / +5% hx | STA | 70 | 4.6 | 0.5 | FII |
| 20190322_5hx_003 | DK428 | 6h / +5% hx | STA | 70 | 4.6 | 0.5 | FII |
| 20190322_5hx_004 | DK428 | 6h / +5% hx | STA | 70 | 4.6 | 0.5 | FII |
| 20190322_5hx_008 | DK428 | 6h / +5% hx | STA | 70 | 4.6 | 0.5 | FII |
| 20190322_5hx_035 | DK428 | 6h / +5% hx | STA | 70 | 4.6 | 0.5 | FII |
| 20190322_5hx_040 | DK428 | 6h / +5% hx | STA | 70 | 4.6 | 0.5 | FII |
| 20190322_5hx_041 | DK428 | 6h / +5% hx | STA | 70 | 4.6 | 0.5 | FII |
|  |  |  |  |  |  |  |  |
| **Projection images** |  |  |  |  |  |  |  |
| DMSO | EW104 | 6h + 2h DMSO | S6 | 14 | 3.4 | 10 | K3 |
| Lat-A | EW104 | 6h + 2h Lat-A | S6 | 14 | 3.4 | 10 | K3 |
| Haploid | LY2 | 6h | S1 | 10 | 7.3 | 0.5 | FII |

The cryo-ET data from this table were deposited as EMPIAR-10670. NKY611 = wild type; DK428 = wild type + Zip1-GFP; EW104 = *ndt80Δ* + Zip1-GFP; NKY2535 = *spo11Δ/spo11Δ, ndt80Δ//ndt80Δ*; NKY2460 = *zip1Δ/zip1Δ*; LGY0068 = *red1Δ0/red1Δ0, ndt80Δ/ndt80Δ*. SM time = duration in sporulation medium. hx = 1,6-hexanediol. STA = subtomogram averaging analysis. Dose, in electrons / Å^2^. For K3 data, pixel size corresponds to the physical “bin ×1” pixels. Nominal underfocus (∆f) values are reported. Volta phase contrast data was acquired at 0.5 µm underfocus. Cam, camera: Falcon II (FII) and K3-GIF (K3).
