# Supplementary material for: Cryo-ET detects bundled triple helices but not ladders in meiotic budding yeast
Source: PLoS One. 2022 Apr 14;17(4):e0266035. doi: 10.1371/journal.pone.0266035 (PMC9009673; doi:10.1371/journal.pone.0266035)
Supplement: S5 Table — (DOCX) [file pone.0266035.s014.docx]

**S5 Table. Template-matching details.**

| **Complex** | **Reference** | **Grid spacing** | **Angular steps †** | **CC cutoff** | **low-pass filter** | **overlap limit** |
| --- | --- | --- | --- | --- | --- | --- |
| MTH | 30-nm × 12-nm (H × D) cylinder | 16 nm | 10° | 0.2 | 60 Å | 11 nm |
| Ribosome | 25-nm diameter sphere | 30 nm | n/a | 0.2 | 60 Å | 30 nm |

CC = cross-correlation. † Owing to the cylindrical symmetry of the reference used for the MTH segments, the angular search was not done around the cylindrical axis and was also limited to ± 90° around the other two axes. No angular search was done for ribosomes because the reference had spherical symmetry. If two template matching hits were positioned within the overlap limit, they were considered duplicates and one was removed automatically in PEET.
