# Supplementary material for: Cryo-ET detects bundled triple helices but not ladders in meiotic budding yeast
Source: PLoS One. 2022 Apr 14;17(4):e0266035. doi: 10.1371/journal.pone.0266035 (PMC9009673; doi:10.1371/journal.pone.0266035)
Supplement: Attachment — Submitted filename: 117289_1_rebuttal_2143532_qxbcw5.docx [file pone.0266035.s015.docx]

**Manuscript number:** RC-2021-00831

**Corresponding author(s):** Lu, Gan

Reviewer comments are in black regular font. Our rebuttal is in **blue bolded font**. Experiments that we plan for the full revision are preceded with “**FULL:**”. In the revision files, the changes are highlighted in yellow.

**1. General Statements**

**We thank the reviewers for their detailed feedback. There are two major concerns. First, the manuscript lacks functional analysis of the meiotic triple helices (MTHs). Second, the manuscript makes claims about the properties of synaptonemal complexes (SCs) and MTHs that are inadequately supported. In order to address the first concern, we would need extensive experiments to first identify and then perturb the genes associated with the MTH. Such experiments are beyond the scope of this manuscript and are the focus of future studies. We will address the second concern with mostly text revisions. We will also improve some of the imaging analysis with new experiments that can be done in a few months’ time.**

**2. Description of the planned revisions**

**We will acquire new cryo-ET data of pachytene-arrested cell cryolamellae using our new K3-GIF camera. These new data have higher signal-to-noise ratios and allow us to generate a higher-resolution subtomogram average of the MTHs. The achievable resolution will depend on the conformational homogeneity of the MTH segments and on the number of cryotomograms we can capture. If we are able to achieve a subnanometer-resolution reconstruction, we will narrow down the possible identities of the subunits. Even if we cannot achieve subnanometer resolutions, the new data will allow us to test if ladder-like densities were missed in our lower-resolution older data, thereby improving our understanding of the SC’s structure. We will also perform subtomogram analysis of purified ribosomes as a control to strengthen our handedness determination.**

**3. Description of the revisions that have already been incorporated in the transferred manuscript**

**The Reviewers’ original comments are reproduced in regular black font. Line numbers refer to the preliminary revision.**

Reviewer #1 (Evidence, reproducibility and clarity (Required)):

Ma and coworkers report studies of budding yeast undergoing meiosis by cryo-ET. They fail to detect structures interpretable as synaptonemal complex, and instead detect feather-like bundles of what appears to be a triple helix. These structures do not appear to be related to the synaptonemal complex, as spo11 mutants that do not initiate recombination, red1 mutants that lack axial elements, and zip1 mutants that lack the central element of the SC still make these bundles. These structures are absent from cells treated with latrunculin A, which depolymerizes actin filaments, but expected structures are not visible in light microscopy of cells treated with two different F-actin-staining reagents. However, it should be noted that another study (Takagi et al, 2021, bioRxiv) did detect actin associated with these structures by immunogold labeling. The structures are also reversibly dissolved in 7% hexanediol.

This part of the paper's findings is well supported by data and is certainly of interest, although interest is somewhat limited by the unknown nature of these structures-what they contain, let alone their function, remains to be determined-in fact, it is not even determined whether or not they are made of protein. However, as an initial report of a previously unknown phenomenon, the paper is of some value.

**Thank you for raising the issue of whether MTHs are composed of protein or not. Aside from the proteins, the only other materials capable of forming large bundles of linear polymers are polysaccharides and DNA. Yeast polysaccharides are found in the cell wall, so they are unlikely to be a candidate for the MTHs. In the nucleus, DNA is abundant. While we favor that MTHs are composed of protein, we cannot rule out that the MTHs are non-chromatin DNA-protein complexes. Depending on the resolution of future subtomogram averages, we may get a better idea of the MTH’s composition.**

There is, unfortunately, a second aspect of the paper that cannot be supported. Although it is clear that synaptonemal complex is present in the cells examined (by standard cytological methods) the authors cannot detect structures consistent with SC in their cryo-ET images. Unfortunately, authors then extrapolate from their inability to detect SC to conclusions about SC, such as that it is not crystalline, and even go so far as to suggest that their failure to detect SC invalidates two models for crossover interference, and that the ladder-like structure reported for SC in many organisms using many difference approaches may be a fixation artifact. Authors show remember that the absence of evidence is not evidence for absence; the speculation described above should be removed from both the abstract and discussion.

**We have removed the speculation about crossover interference and limited the scope of our discussion on SCs to budding yeast only.**

**The differences between traditional EM and cryo-EM are not trivial. In the introduction, we added more details to explain the differences in both the sample preparation and contrast generation:**

**Lines 79-87: “Meiotic nuclei have been studied for decades by traditional EM (Fawcett, 1956; Moses, 1956), but not by cryo-ET. Cryo-ET can reveal 3-D nanoscale structural details of cellular structures in a life-like state because the samples are kept unfixed, unstained, and frozen-hydrated during all stages of sample preparation and imaging (Ng and Gan, 2020). The densities seen in cryo-ET data come from electron scattering of the biological macromolecules. In comparison, the densities seen in traditional EM from electron scattering of heavy metals such as uranium, tungsten, and osmium, which have adhered to a subset of biological macromolecules that were not extracted in earlier steps.”**

**We have also removed the term ‘artifact’ from lines 201-202:**

**Original: “The ladder model is based on images of traditional EM samples, which are vulnerable to fixation and staining artifacts.”**

**Revised: “The ladder model is based on images of traditional EM samples.”**

Reviewer #1 (Significance (Required)):

This paper reports a previously unreported structure in the nuclei of yeast cells undergoing meiosis. The composition and function of this structure remain to be determined. This considerably limits the significance of the paper.

**Referee Cross-commenting**

I agree with the concerns of the other reviewers. I also agree with reviewer 3 that to raise the significance of the paper would require much work. But I think that the raw observation is of value, albeit in a journal of record. So I would stick with my recommendation of text changes, keeping in mind that there may not be a suitable journal in LSA's portfolio.

Reviewer #2 (Evidence, reproducibility and clarity (Required)):

This work describes helical filamentous structures observed in budding yeast nuclei that were cryosectioned and imaged using cryo-electron tomography (cryoET).

The goal of this work seems to have been to conduct an ultrastructural analysis of the synaptonemal complex (SC), a meiosis-specific protein structure that holds chromosomes together during meiosis and is thought to regulate meiotic recombination. In conventional TEM images of fixed, embedded, and stained sections obtained from pachytene nuclei, SCs usually appear as long, thin, transversely striated structures. At pachytene, SCs extend along the full length of a thin (100-nm) gap between paired chromosomes (typically 1-6 µm long in yeast cells). Surprisingly, the authors did not observe SCs, perhaps because these structures do not produce much contrast in cryo-EM images. Instead, they observe abundant triple helical structures in the nucleoplasm, which they designate as "meiotic triple helices" (MTHs). The authors report that these triple helices assemble at the same time as but independently of the SC. They publised a preprint in which they indicated that these structures were somehow related to SCs, but this the revised version reports that they appear independently of SCs. They further report that treatment of cells with the F-actin depolymerizing drug Latrunculin-A (LatA) resulted in a lack of detectable triple helical structures in the nucleus, suggesting that these structures may be a form of actin or, alternatively, that they may require F-actin for their assembly.

While the work is technically mostly sound, its significance is unclear because the reported structures have no known function. Many, if not most proteins will form helical structures if their concentration rises above a threshold defined by their binding affinity for themselves (see https://doi.org/10.1186/1741-7007-11-119 and references therein), so this may simply be an example of an abundant nuclear protein that polymerizes to form helical filaments under the conditions that trigger yeast sporulation.

**Thank you for raising the interesting possibility that MTHs form helices because their subunits have exceeded a critical concentration threshold. In the revised text, we discuss the possibility that the MTH is simply a protein that is highly expressed in meiotic cells and polymerize either due to exceeding a critical concentration, or having undergone a biochemical change like a post-translational modification:**

**Lines 408-415: “Note it is possible that the MTHs may not be directly involved in meiosis, but are instead a protein that is at a sufficient concentration or has the right biochemical modifications to form helices in pachytene because it is known that many proteins can form a helix under the right conditions {Crane, 1950; Pauling, 1953; Theriot, 2013}. These MTHs also have lateral interactions that allow them to pack with crystalline density. Their sensitivity to 1,6-hexanediol suggests that the polymerization both within and between MTHs are based on hydrophobic interactions. Further work will be needed to determine the identity of the MTH’s subunits and their potential function.”**

The authors perform cryosectioning and cryoEM on yeast cells undergoing meiosis to show that the assembly and disassembly of MTHs follow a similar time course as that of the SC. The observation of these triple-helical filaments in meiotic nuclei has also been reported in another study (https://www.biorxiv.org/content/10.1101/778100v2.full.pdf+html), which proposed, based on immuno-EM labeling, that they may be actin cables. This study reports that the structures are not detected using phalloidin or Lifeact-mCherry. However, treatment with LatA did eliminate detection of MTH structures, suggesting that they may be comprised of actin.

In my view, there are a number of issues that should be addressed before publication. Many of these relate to the presentation of the findings. Detailed comments below:

1. The presentation of the work is very confusing. The authors clearly expected to observe SC structures, but did not. They conclude that MTHs are not SCs, since they do not depend on molecular components required for SC assembly. They should describe their findings in a more straightforward way rather than veering from introducing the SC to describing the MTHs.

**We have restructured the manuscript to tone down the discussion about the SC. However, we have to start with the SC because it is the most iconic feature of pachytene cells and a major organizer of chromatin in meiosis. Furthermore, its presence, as indicated by Zip1-GFP signals, was key to establishing that our cells were indeed arrested in pachytene. It would have been confusing to then overlook the absence of structural features as conspicuous and prevalent as what was expected of SCs. The other sections did have room for improvement. In the sections below, we describe the changes point by point.**

Similarly, the discussion section on "recombination and chromosome segregation" seems inappropriate and irrelevant, since no data are presented in this study regarding the functions of the MTHs, and there is no reason to think that they contribute to crossover interference, chromosome segregation, or other aspects of meiosis. Additionally, most of the ideas presented in this section seem very muddled. I recommend deleting this section.

**We have deleted the section entitled "Recombination and chromosome segregation".**

**Throughout the text, we have also changed the term “meiosis-specific” to “meiosis-related” when describing MTHs. Doing so allows for the possibility that MTHs might just form as a consequence of being expressed to a high enough concentration as discussed above.**

2. Along the same lines as comment #1: The title should be changed - absence of evidence for "ladders" is not evidence of absence. Prior work using TEM and superresolution fluorescence microscopy has clearly shown that ladder-like SC structures exist in pachytene nuclei of budding yeast and many other organisms, although they apparently cannot be visualized using the methods described here.

**We have changed the title to be less forceful, yet report what we see and don’t see by cryo-ET:**

**“Cryo-ET detects bundled triple helices but not ladders in meiotic budding yeast”**

3. The authors should clarify whether cryo-sectioning was performed through the full volume of pachytene nuclei.

**This comment refers to our attempt at serial cryosectioning, as shown in Fig. S8. We have revised the text in lines 332-334 to reflect the estimated volume covered:**

**“We successfully reconstructed six sequential sections from one ndt80Δ cell (Figure S8), which represents approximately one third of a nucleus (assuming a spherical shape).”**

**We also changed Fig. S8’s title so that it doesn’t sound like we reconstructed an entire nucleus:**

**“MTH bundles are extensive throughout the cell nucleus.” → “MTH bundles are extensive.”**

4. It is not clear from the manuscript which camera/microscope configuration was used to acquire the cryoET data that were used for sub-tomogram averaging. The authors state in the methods that Falcon II and K3-GIF was used for projection images, but it's not clear if this applies to all images. These technical details should be clarified.

**We have added a new column to Table S4 that reports the camera used for all the projection imaging and tomography experiments. In the original MS, all of the subtomogram averaging was done using Falcon II data.**

**FULL: In the full revision, we plan to incorporate new subtomogram averages of MTHs in situ, using K3-GIF data of cryolamellae.**

5. The analysis of the handedness of the helices seems to be questionable as the resolution of the reconstructions for 80S are also quite low. I am uncertain whether this can be used to state with confidence that the MTH are right-handed.

**FULL: In the full revision, we will use purified 70S ribosomes, imaged on the same K3-GIF camera and using the same software workflow as for the new subtomogram averaging of MTHs in situ. We expect higher resolution for both ribosomes and MTHs, which will make the handedness assignment unambiguous.**

6. The authors claim that treatment with Latrunculin-A (LatA) leads to disappearance of MTHs. However, they support this with projection images of cells treated with LatA. The projection images are of poor quality and the vitrification in these cells (as well as the DMSO treated cells) do not look appropriate. They should present data for LatA-treated cells and DMSO-treated controls obtained using the same approach and ideally imaged in parallel with untreated cells. They should also quantify the number of sections and cells imaged for all conditions.

**Once we realized that the MTH bundles were visible in projections, we chose to report detections of MTH bundles by projection imaging instead of the costly tilt series. The apparent poor quality and questionable vitrification comes from the fact that the projection images show the cryosection’s crevasses and knife marks, which reside on opposite cryosection surfaces. These sectioning artifacts are computationally excluded from tomographic slices. The following line was added to the figure caption to explain this:**

**“These image features are not devitrification artifacts; they are absent from the tomographic slices in other figures because they can be computationally excluded.”**

**The quantification of the MTHs in Lat-A vs control cells are in Table 2. We have now added these numbers to both the text and the figure caption.**

7. The similarities between the MTHs and SCs - that both are present in meiotic nuclei and sensitive to hexanediol - seem unlikely to be functioanlly relevant. Again, I think the presentation suffers from being focused on the SC which was not seen, rather than on the MTHs.

**We have toned down the discussion on SCs throughout the manuscript. We have retained the motivation for using 1,6-hexanediol to probe the MTHs physico-chemical properties and the fact the previous work on SCs provided motivation for this perturbation experiment. However, we removed the comparison of their relative sensitivities to 1,6-hexanediol (see reply to point #10 below).**

8. The absence of 100-nm-wide zones containing nucleosomes is again not evidence for lack of SCs. SCs are ribbon-like structures - they are about 100 nm in one dimension but the thickness has not been characterized reliably; even if SCs do exclude nucleosomes (which is not certain) the excluded volume might be much smaller than the authors imagine.

**We did not argue for “lack of SCs”; these structures clearly exist in our cells given the fluorescent linear structures seen in Zip1-GFP expressing cells. We only say that the textbook portrayal of SCs needs revision, though we should have restricted our statement to yeast. In the literature and textbooks: whenever the SC’s central element is drawn, it is depicted without internal nucleosomes and being densely packed with SC proteins.**

9. Does Lifeact-mCherry enter the nucleus? This information is important in interpreting the failure to detect MTHs using this probe.

**While Lifeact-mCherry is small enough to passively diffuse through the nuclear pore, our data cannot rule out that this molecule is excluded from the nucleus. We added the following sentence as a caveat:**

**Lines 244-245 “Note that we cannot rule out that Lifeact-mCherry is excluded from the nucleus.”**

10. The sensitivity of the MTH structures to 1,6-hexanediol treatment is potentially interesting, but it does not reveal anything about their structure or function, only that their assembly likely depends in part on hydrophobic interactions. Caution should be used in interpreting these findings.

**We have toned down the discussion about the meaning of the MTH bundles’ 1,6-hexanediol sensitivity by removing this line from the original Results:**

**“MTH bundles are therefore sensitive to a slightly higher concentration of 1,6-hexanediol than SCs are and reform when 1,6-hexanediol is removed.”**

**We have also added the following line to more clearly say what sensitivity to 1,6-hexanediol means:**

**Lines 412-414: “Their sensitivity to 1,6-hexanediol suggests that the polymerization both within and between MTHs are based on hydrophobic interactions”**

11. The figure legends and/or Methods sections should clarify what is represented in each figure, and how the data were acquired. In particular, cryotomographic slices of varying dimensions (6nm, 10 nm or 12 nm or 70 nm) are mentioned in the captions of several figures (2-6, and S1, S2, S3). However, is often unclear whether these represent physical or computational sections.

**“Tomographic slice” refers to a rendering of a computationally extracted slice from a reconstructed tomogram. To make it clearer, we have added the term “computational” to describe the tomographic slices in each figure caption.**

12. Page 23 has a supplemental figure but no captions. Is this the same as Fig. S8?

**Yes, this is a copy of Fig. S8 that appeared due to MS Word’s jumping-figure bugs. We will manually edit the PDF in the future revision.**

13. I do not find the model figure (Figure 7) to be helpful. Additionally, the failure to detect SCs and the presence of MTHs do not warrant a "revised model of the meiotic yeast nucleus."

**We now call panel A and B the “Traditional EM” and the “Cryo-EM” models, respectively. The figure therefore reports the large nuclear bodies seen by the two methods and no longer implies correctness.**

**We also changed the related sentence in the Introduction:**

**Original: “Our work strongly suggests that current models of pachytene nuclear cytology need revision.”**

**Revised: “Our analysis shows that MTHs coexist with SCs, which have an unknown cryo-ET structure.”**

14. The absence of MTHs in haploid cells induced to undergo meiosis should perhaps be studied in more detail. Even SCs are present in haploid meiotic cells, so the absence of these structures may be informative as to their function. Haploid cells should also be stained for SCs and imaged by immunofluorescence to verify that they are in meiosis.

**The haploid strain that was treated with sporulation media cannot enter meiosis. Haploid cells that are capable of entering meiosis need to be disomic for chromosome III, with each copy having a different mating type at the Mat locus. We believe that the construction and studies of such strains would be more meaningful after we identify the MTH’s subunits and determine its function in diploid cells.**

15. The yeast strain is SK1, not SK-1.

**Thank you. This mistake is corrected.**

16. What are "self-pressurized-frozen samples" (p.2)?

**Self-pressurized-frozen samples are generated by an alternative to the conventional machine-based high-pressure-freezing method. We have added more details in the new lines 135-141:**

**“Self-pressurized freezing is a simpler and lower-cost alternative to conventional high-pressure freezing, which requires a dedicated machine that consumes large amounts of cryogen. In the self-pressurized freezing method, the sample is sealed in a metal tube and rapidly cooled in liquid ethane. The material in direct contact with the metal cools first and expands by forming crystalline ice, which exerts pressure on the material in the center of the tube (Leunissen and Yi, 2009; Yakovlev and Downing, 2011; Han et al., 2012).”**

Reviewer #2 (Significance (Required)):

The observation of MTHs is novel but (as stated above) of unclear significance, given that their molecular identity and function are unknown.

This work may be of interest to the meiosis field, with the caveats described above that the functional relevance is currently unclear.

This review was co-written by referees with expertise in meiosis, chromosome organization, SC structure and function, and cryoEM.

**Referee Cross-commenting**

The reviews are strikingly concordant so I don't think much needs to be added.

Reviewer #3 (Evidence, reproducibility and clarity (Required)):

The authors report the observation of filamentous structures (further termed meiotic triple helices MTH) in the nucleoplasm during meiosis in yeast cells. Those structures are visualized using Cryo-ET. While the authors initially seem to assume an association of those structures with synaptonemal complexes, they discover that those structures rely on filamentous actin and are not affiliated with synaptonemal complexes after all.

While I think that the observation of those MTH by Cryo-ET is interesting, the overall structure of the paper and presentation of the data are not very well done. As the authors find throughout their experiments that the MTHs are not associated with synaptonemal complexes the strong focus in the first figures on synaptonemal complexes as well as the title of the paper are very mis-leading. The authors try to initially make the point that other labs have observed ladder like structures by transmission electron microscopy and want to make the claim that those observed structures might be an artifact of sample preparation, hence the title: Meiotic budding yeast assemble bundled triple helices but not ladders. However, at the end those structures seem unrelated to synaptonemal complexes.

In addition, several labs have reported the presence of nuclear actin in meiosis and mitosis and have even succeeded to show those structures by transmission electron microscopy, questioning the "artifact" argument.

**Presumably, the Reviewer means intranuclear actin, as opposed to perinuclear (cytoplasmic) actin. If so, then we have only seen one paper, the one from the Takagi et at 2021 (bioRxiv) that has reported seeing structures associated with nuclear actin. Note however, that the revised Takagi bioRxiv paper is very careful in saying that the filament bundles contain actin, which is not the same as saying that the filaments are polymers of actin.**

**Our “artifact” argument – now removed – referred to SCs, not to nuclear actin.**

**In the revised manuscript, we use the term “intranuclear” to make it clear that we are referring to structures inside the nucleus. We also use the term F-actin, where appropriate, to refer to the best-studied actin polymer, which resembles a double helix. Doing so eliminates confusion about other forms of actin: G-actin, which cryo-ET cannot yet directly visualize due to its small size; and non-canonical actin polymers, for which there are no previous experimental X-ray or cryo-EM structures for comparisons.**

The story line of the paper is weak and I think the authors would have been better of reporting their cryo-ET structures and making a better link to actin or determining what else they think might be a component of those structures. Immuno-EM (as actually shown in Reference 41) of actin would have been much more convincing. The authors could also use the power of Cryo-ET and the achievable higher resolution to describe those filaments in much more detail. In my opinion this would have been a much better and more exciting paper.

**We disagree that “making a better link to actin” is the right approach because doing so presupposes that the structures are composed of actin, for which the present evidence is inadequate. We do agree that determining what the MTHs are (or are not) would be valuable.**

**FULL: Now that we have better access to a K3-GIF camera and a cryo-FIB-SEM, we will attempt higher-resolution subtomogram averaging analysis. If we are able to achieve subnanometer resolution, we will attempt to narrow down the fold of the MTH subunit. Note that this goal will require that the MTHs are conformationally homogenous and that we can image sufficient copies of the MTHs.**

In summary: While I think the Cryo-ET images of those structures could be very exciting the paper unfortunately does not do a very good way in presenting this data and is at times misleading trying to proof or rather dis-proof a connection to synaptonemal complexes. Based on this I think that the paper can not be published in the current form and needs major revisions that would require a significant amount of time.

**Minor comments:**

Figure 1: The choice of timepoints is confusing and makes it hard to compare. While wild type is shown at 0,1,3,4,5,8h, the mutant is shown at 0,2,3,4,5,6,7h. It would be appropriate to select the same timpepoints for both conditions.

**FULL: We will recollect fluorescence images of the mutant cells at the same time points as the wild type.**

Figure 3 and 4 need a quantification of the number of observed MTHs, in particular as only selected regions of the nuclei are shown.

**These images were taken from single cryosections instead of serial cryosections, which would have been too difficult to do for multiple conditions and multiple cells. Therefore, quantification would be obfuscated by the fact each cryosection samples a small fraction of the nuclear volume. We believe that reporting the number of cell cryosections that are MTH-positive (Table 2) is at present the best way to characterize their abundance and ability to polymerize. Once we are able to identify the MTH gene products, we will be able to perform GFP tagging experiments and thereby get a much better estimate of the polymer mass as a function of biochemical perturbations.**

Fig 7. The data certainly does not support a "REVISED" model of the yeast nuclear organization.

**We have changed the Figure title to “A cryo-ET model of the meiotic yeast nucleus.” We now also refer to panels A and B as “Traditional EM model” and “Cryo-ET model”, respectively.**

Reviewer #3 (Significance (Required)):

Several publications have already shown the presence of actin in meiotic and mitotic nuclei and have even succeeded in observing those structures by transmission electron microscopy. Based on this it is not clear why the authors have not tried to put their work in context to all these observations and used their technology to obtain novel information on the structure, which might be helpful to identify which proteins compose the MTHs. Based on how the data is presented I do not think that this paper contributes anything new to the field.

**Presumably, the reviewer means that F-actin has been imaged in yeast cells, because for actin to exist in nuclei in mitosis/meiosis, the organism would have to undergo closed mitosis/meiosis. Furthermore, for actin to be observable by transmission electron microscopy, it would have to be in the filamentous (F-actin) form. We could not find any publications that report transmission electron microscopy of F-actin in yeast cells. We therefore cannot relate our results to F-actin in the meiotic nucleus.**

My field of expertise is meiosis and mitosis as well as imaging (light and electron microscopy).

**Referee Cross-commenting**

All reviewers seem to agree that the general observation of these structures is interesting but that there is a reduced significance as the function and identity of these structures remains unknown.

**4. Description of analyses that authors prefer not to carry out**

**The main unanswered question is: what is the function of the MTH bundles? To address this question, we would first need to identify the gene products that are needed for MTH assembly. Next, we would need to do genetic perturbation experiments to actually determine the MTHs’ function. These experiments would constitute a complete study, which is better suited for a separate, future manuscript.**
